# An Illustrative Analysis of Atypical Gas Production Profiles Obtained from In Vitro Digestibility Studies Using Fecal Inoculum

**DOI:** 10.3390/ani11041069

**Published:** 2021-04-09

**Authors:** Mewa S. Dhanoa, Secundino López, Christopher D. Powell, Ruth Sanderson, Jennifer L. Ellis, Jo-Anne M. D. Murray, Anna Garber, Barbara A. Williams, James France

**Affiliations:** 1 Department of Animal BioSci.s, University of Guelph, Guelph, ON N1G 2W1, Canada; dandhanoa@hotmail.com (M.S.D.); cpowell@uoguelph.ca (C.D.P.); jellis@uoguelph.ca (J.L.E.); 2 Departamento de Producción Animal, Universidad de León, 24007 León, Spain; 3 Instituto de Ganadería de Montaña, CSIC-Universidad de León, Finca Marzanas s/n, 24346 Grulleros, Spain; 4 Institute of Biological, Environmental and Rural Sciences, Aberystwyth University, Aberystwyth SY23 3EB, UK; rts@aber.ac.uk; 5 College of Medical, Veterinary and Life Sci.s, School of Veterinary Medicine, University of Glasgow, Glasgow G61 1QH, UK; Jo-Anne.Murray@glasgow.ac.uk (J.-A.M.D.M.); a.garber.1@research.gla.ac.uk (A.G.); 6 Centre for Nutrition and Food Sciences, Queensland Alliance for Agriculture and Food Innovation, The University of Queensland, St. Lucia, QLD 4072, Australia; b.williams@uq.edu.au

**Keywords:** gas production technique, feedstuff evaluation, Mitscherlich equation, atypical profiles, numerical modeling, equivalent profile construction

## Abstract

**Simple Summary:**

The gas production method is a laboratory technique that measures the amount of fermentation gases produced from incubating animal feedstuffs with microbes from ruminal fluid or fecal samples. It is faster and cheaper than evaluating feedstuffs using animal trials. Models may be applied to the gas production profiles generated in order to rank feedstuffs or to determine the extent of feedstuff digestion either in the rumen or in the hindgut. Typical gas production profiles show a monotonically increasing monophasic pattern. However, atypical gas production profiles exist whereby at least two consecutive phases of gas production or additional extraneous features which distort the typical profile are present. Such profiles are more likely to occur with the use of a fecal inoculum and are much less well described. This article illustrates the analysis of these atypical gas production profiles and explores the methodology of numerical modeling to construct equivalent typical growth-like trends.

**Abstract:**

Gas production profiles typically show a monotonically increasing monophasic pattern. However, atypical gas production profiles exist whereby at least two consecutive phases of gas production or additional extraneous features that distort the typical profile are present. Such profiles are more likely to occur with the use of a fecal inoculum and are much less well described. The presence of multiple phases or non-descript extraneous features makes it difficult to apply directly recommended modeling approaches such as standard response functions or classical growth functions. To overcome such difficulties, extensions of the Mitscherlich equation and a numerical modeling option also based on the Mitscherlich are explored. The numerical modeling option uses an estimate of relative rate obtained from the smoothed data profile and an estimate of maximum gas produced together with any lag time information drawn from the raw data to construct a simple Mitscherlich equation. In summary, this article illustrates the analysis of atypical gas production profiles obtained using a fecal inoculum and explores the methodology of numerical modeling to reconstruct equivalent typical growth-like trends.

## 1. Introduction

For the success and sustainability of animal production enterprises, proper feeding and adequate nutrition of the animals are paramount. To achieve this goal, it is necessary to establish the nutritional quality of animal feedstuffs. For this purpose, the early method mainly used was whole-tract in vivo digestibility; digestibility being the proportion of feed ingested actually utilized by the animal. To determine digestibility, one needs to measure intake and voided fecal matter. Animals are usually housed in individual crates because of the need to assess total amounts of any refusals and fecal matter, and that limits the capacity of such experiments and interferes with the animal’s ingestive processes as well as being labor intensive. In order to apply the 3R principles (reduce, replace, refine) in animal experimentation, in vitro methods appear to be a suitable alternative for estimating digestibility and for general feed characterization purposes. The need for standardized inoculum meant the use of a few donor animals kept and fed indoors, and thus devoided of free grazing behavior. To avoid any natural behavior constraints and to cover non-domesticated species, more sources of inocula, including voided feces, also became popular [1,2]. Despite some differences (e.g., in lag-time and half-life), results from inoculum derived from feces tend to be well correlated with those from rumen liquor or cecal fluid [3,4].

**Figure 1:**
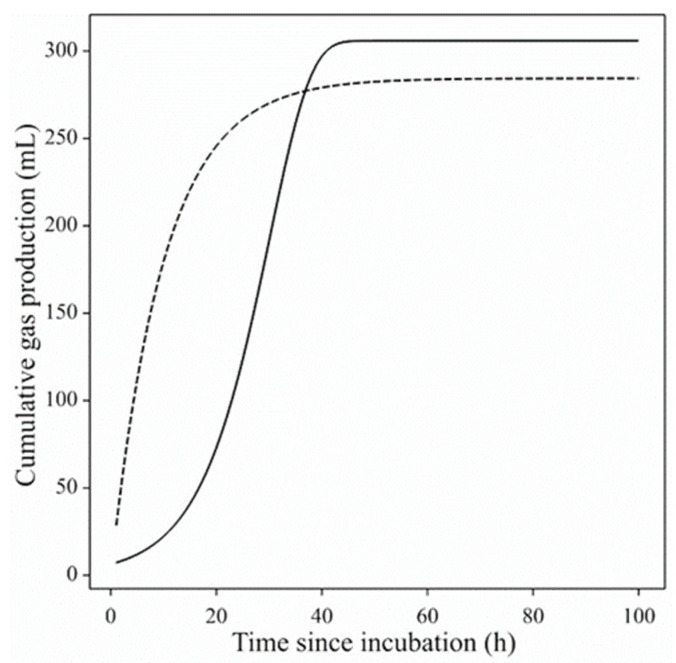
Typical gas production profiles (Mould et al. [13]). The fitted profiles were produced using 200 mg of a pure substrate [glucose (dashed line) and cellulose (solid line)] and 10 mL of a standard rumen fluid inoculum.

Cumulative in vitro gas production (GP), being proportional to substrate degradation, has become a surrogate for in vivo digestibility and GP profiles are expected to exhibit an asymptotic shape with or without an inflexion point. Profiles without an inflexion point tend to follow a diminishing returns pattern. Simple substrate fermentation usually leads to a single-phase shape whilst a more complex substrate might lead to biphasic or other shapes [5,6,7]. Similarly, a well-defined inoculum such as rumen fluid should lead to the shape expected for the experimental substrate. Typical GP profiles are illustrated in Figure 1. Fecal inocula tend to be increasingly used because of easiness and animal welfare issues, but the classical models most commonly used to fit the GP curves are not always suitable to describe curves that do not follow the typical pattern. When equine feces are used as inoculum source, expected standard shapes may not always be the result [8]. In such a setup, substrate and inoculum interactions can lead to additional extraneous features which might distort the GP profile. Any treatments applied to the substrate or the inoculum may add further complexity. Gas production profiles generally represent three stages, the lag stage (slow GP rate), the exponential stage (fast GP rate), and the stationary stage (diminishing GP rate). However, sometimes the curves show deviations from this typical pattern, exhibiting non-steady increasing profiles with bumps or steps (as if fermentation occurred in sequential waves) or without a clearly defined upper asymptote after long incubation times [8]. Some multiphasic or multistage models have been proposed to deal with complex substrates containing fractions that are fermented at clearly differentiated rates [5,6,7]. However, even in those cases, the GP profile followed a more regular pattern, whereas when fecal inocula are used it is usual to observe irregular profiles that have not been sufficiently studied. With increasing interest in the use of such fecal inocula, more research is required exploring alternatives to provide insights and suitable solutions to estimate fermentation parameters from atypical GP curves.

In manual or semi-automatic systems [9,10,11], headspace is often emptied at some pre-set pressure level, e.g., in the manual systems a 4–7 psi level is generally appropriate. Users of the ANKOM RF Gas Production System (ANKOM Technology, Macedon, NY, USA) [12] appear to use a fixed time interval of usually 5, 10, 15, or 20 min to actually record GP data whilst accumulating data internally at some lower psi level. However, this can create a problem during the exponential stage. The GP profiles can seem disjointed. The study of simple GP rate (d*G*/d*t*, where *G* is cumulative GP (mL) at incubation time *t* (h)) becomes difficult and non-informative. Thus, if we have non-standard or contaminated GP profiles, the modeling may have to change to make further progress, i.e., some kind of data pre-processing may possibly be needed.

Thus, the primary objectives of this study are (i) to illustrate the analysis of atypical GP profiles and (ii) explore the methodology of numerical modeling by constructing equivalent standard growth-like trends. For this latter purpose, data smoothing, appropriate mathematical equations, and numerical calculations such as fractional or relative rates are required. This work is based on the Mitscherlich equation, a response function with a constant relative rate discussed extensively in the GP context by France et al. [14] and Powell et al. [8].

## 2. Materials and Methods

### 2.1. Datasets and Gas Production Technique

The data utilized in this study were taken from three studies. The first study provided data obtained from two GP trials undertaken at the University of Queensland using the same experimental protocol [1]. The first trial investigated a range of fruit and vegetables (viz. apple, banana, carrot, celery, pear, spinach, and wheat bran) [15]. The second trial examined a range of nuts and legumes (viz. almond (coarse particles), almond (fine particles), chickpea (fine), lentil (fine), macadamia (coarse), macadamia (fine), mung bean (fine), peanut (coarse), peanut (fine), and wheat bran (control)) [16]. The cumulative GP technique was used as described by Williams et al. [1], employing an automated gas recording system [17]. Five replicates of each substrate were fermented using porcine fecal inoculum and gas readings were taken at regular intervals over 48–72 h. The feces were collected from five male Large White grower pigs of 30–35 kg that had consumed a standard semi-defined diet, based on maize starch and fishmeal, for at least 10 d prior to collection. The diet was formulated to be as free as possible of potentially fermentable carbohydrates to prevent adaptation of the microbial population. Cumulative gas production was measured as a function of time and corrected to the volume (mL) of gas produced per gram of substrate dry matter. The study yielded 17 atypical GP profiles, one for each substrate, as the average over the five replicates.

The second study from horses [18] assessed the fermentative capacity of fecal inocula sourced from 14 grass-kept horses (maintained on grass 24 h a day) from the International League for the Protection of Horses in Norfolk, UK. Inocula were prepared from these 14 horses—7 of them predisposed to laminitis and the other 7 clinically normal—so that the effect of laminitis on hindgut fermentative activity could be evaluated. Grass hay was one of the substrates incubated in vitro. Gas production was recorded manually using the method of Theodorou et al. [11] and three replicates per inoculum were used. Standard in vitro GP results were described by Murray et al. [18]. The grass hay data yielded 14 (predominantly atypical) GP profiles, one for each horse, as the average over the three replicates.

The third study [19] comprised a total of 11 different fecal inocula. Eight of these inocula were sourced from 8 Welsh Section A geldings arranged in a double 4 × 4 Latin square experimental design aiming to investigate the in vitro fermentation of high fiber/high concentrate diets supplemented or not with yeast (control diets with no yeast). Another 3 fecal inocula were obtained in an experiment in which ponies were fed a grass hay only diet (control), or the same grass hay supplemented with increasing concentrations of a fibrolytic enzyme (either 0.75 or 3.75 mL of enzyme solution per kg DM hay). Three replicates per inoculum were used. Gas production was recorded using the ANKOM GP system [12], designed for measuring the kinetics of a microbial fermentation automatically by monitoring the gas pressure within each individual culture bottle and recording the data remotely in Excel spreadsheets (MS Excel, version 2019, Microsoft, Seattle, WA, USA). The system includes up to 50 individual modules (bottles) that communicate information to a computer using radio frequency transmission through a base coordinator. In the study reported by Garber et al. [19], the global pressure release through internal valves was pre-set to 8 psi, computer communicated to the modules every 10 s, whilst the recording intervals were pre-set for 10 min. The ANKOM system captures data at every tripping but sends out data to be recorded only at set time intervals. Although this avoids excessive pressure building in the headspace, it creates discrete data jumps when recording data only at fixed time intervals. The data yielded 11 GP profiles (showing both typical and atypical patterns), one for each treatment, after averaging the three replicates for each inoculum. A more detailed summary of the data taken from the second and third studies can be found in Powell et al. [8].

### 2.2. Curve Smoothing

Fermentation rates differ at various stages of incubation time. Using the same time interval will create unequal gas amounts due to slow or fast fermentation. Thus, GP data profiles could lack continuity and any subsequent numerical calculations might tend to be non-homogeneous. It would be perhaps better to record GP data more frequently, so as to give a smoother cumulative GP curve. At the modeling stage, the data could then be sampled at suitable time intervals. To make reliable progress in such situations, data smoothing may become necessary in order to undertake the curve fitting. A profile can be smoothed by using smoothing splines regression with large degrees of freedom [20]. Without smoothing, the distribution of values of the GP rate would have some extremes whilst after smoothing the values would be much more as expected. This smoothing can also be achieved by using the autoregressive model AR(*p*). For GP profiles, *p* = 1 is generally sufficient to give the first-order model AR(1), namely:

$$G_{i}=\phi_{0}+\phi_{1}G_{i-1}+\varepsilon_{i}.$$

Here, *G_i_* is the cumulative gas value at time *t_i_*, *G_i−_*_1_ is the gas value one-time step previously, ***ϕ***_0_ and ***ϕ***_1_ are the coefficients of the autoregressive equation (intercept and slope respectively), and *ε_i_* is white noise (residuals from the current model).

### 2.3. Mathematical Considerations

Gas production profiles generally are similar in shape to growth functions (Figure 1). Classical growth functions may have an inflexion point or not (e.g., logistic or monomolecular). An inflexion point may be fixed or variable (e.g., logistic or Richards). There are several other functions suited for modeling growth [21]. In analyzing GP data, rather than adopting or searching for an appropriate growth function, France et al. [14] constructed a new purpose-built function that incorporated all the above features. The function of France et al. [14] is such that the fractional or specific rate (*μ*, h^–1^) varies with time according to the equation:

$$\mu \;=\;b\;+\;ct^{\lambda },$$

where *b* (h^−1)^ and *c* (h^−^λ) are fractional rate constants. From a range of values of *λ*, it was found that *λ* = −1/2 led to a well-behaved function which gave a good fit to the data. Therefore, the expression $b+c/\left(2\sqrt{t}\right)$ was substituted for *μ* in the differential equation:

$$\frac{1}{\left(A-G\right)}\frac{dG}{dt}\;=\;\left(b\;+\;\frac{c}{2\sqrt{t}}\right),\;t\geq T,$$

where *A* (mL) is the asymptotic value of *G* and *T* (h) is the lag time assumed to occur before degradation commences. The conditions *b* ≥ 0, $c\geq -2b\sqrt{T}$ must be satisfied as *μ* cannot be negative. Integrating the above equation gives a generalized Mitscherlich equation [14]:

(1) $$G=A\left\{1-\exp\left[-b\left(t-T\right)-c(\sqrt{t}-\sqrt{T}\right]\right\}.$$

This equation for *G* can be transformed and rewritten as:

(2) $$G=A-BQ^{t}Z^{\sqrt{t}},$$

where $B=Ae^{\left(bT+c\sqrt{T}\right)}$, $Q=e^{-b}$, and $Z=e^{-c}$. At time, *t* = *T* accumulated GP is zero, i.e., $A=BQ^{t}Z^{\sqrt{t}}$. Therefore, lag *T* can be obtained algebraically by solving the quadratic equation in $\sqrt{T}$, i.e., $T\ln Q+\sqrt{T}\ln Z+\ln\left(B/A\right)=0$, giving:

(3) $$\sqrt{T}=\frac{\left\{-\frac{\ln Z}{2}\pm \sqrt{\frac{{\left(\ln Z\right)}^{2}}{4}-\left[\left(\ln\frac{B}{A}\right)\times \ln Q\right]}\right\}}{\ln Q}.$$

Plus or minus root is taken if the estimate of *T* is consistent with the data.

An important special case of this generalized function is the simple Mitscherlich equation [22] occurring when the shape adjustment factor $Z^{\sqrt{t}}\to 1$ (i.e., when c→0): $G=A-BQ^{t}$ with a constant fractional rate. Now the lag is clearly defined where the fitted curve intersects the time axis, i.e., T=ln(A/B)/lnQ. Thus, knowing *A*, *T*, and *µ*, the simpler curve $G=A-BQ^{t}$ is fully described. This means one can construct a representative simple Mitscherlich curve by obtaining estimates of GP asymptote (*A*, mL), lag time (*T*, h), and fractional rate (*b*, h^−1^) from any GP profile modeled with an appropriate mathematical equation. 

Powell et al. [8] derived four models based on the Mitscherlich equation, namely the (i) simple Mitscherlich (Equation (4)), (ii) generalized Mitscherlich (Equation (1) above), (iii) double Mitscherlich (Equation (5)), and (iv) Mitscherlich + linear (Equation (6)):

(4) $$G=A\left(1-e^{-b\left(t-T\right)}\right),t\geq T,$$

(5) $$G=A_{1}\left(1-e^{-b_{1}\left(t-T_{1}\right)}\right)+A_{2}\left(1-e^{-b_{2}\left(t-T_{2}\right)}\right),\;t\geq T_{1},\;t\geq T_{2},$$

(6) $$G=A\left(1-e^{-b\left(t-T_{1}\right)}\right)+\beta \left(t-T_{2}\right),t\geq T_{1},\;t\geq T_{2},$$

to describe four GP patterns: (i) monophasic but diminishing returns, (ii) monophasic but sigmoidal, (iii) biphasic and asymptotic, and (iv) biphasic but non-asymptotic, respectively. The parameter *β* (mL h^−1^) in Equation (6) is the slope of an underlying linear trend; other parameters and variables are as defined above. The four models were fitted directly without transformation, i.e., in the forms presented in Equations (1) and (4)–(6), and extent of digestion was calculated using the fitted parameters [8].

For further investigation and to allow a little more flexibility, a transformed double Mitscherlich equation comprising a generalized Mitscherlich term ($B_{1}Q_{1}{}^{t}Z^{\sqrt{t}}$) and a simple Mitscherlich term ($B_{2}Q_{2}{}^{t}$), was included in this study:

(7) $$G=A-B_{1}Q_{1}{}^{t}Z^{\sqrt{t}}-B_{2}Q_{2}{}^{t}.$$

Additionally, a simplified transformed double Mitscherlich was derived by setting *Z* = 1 in Equation (7), giving:

(8) $$G=A-B_{1}Q_{1}{}^{t}-B_{2}Q_{2}{}^{t}.$$

All data analyses were undertaken using the Genstat statistical software [23].

## 3. Results

### 3.1. Gas Production Curves Using Porcine Fecal Inocula

**Figure 2:**
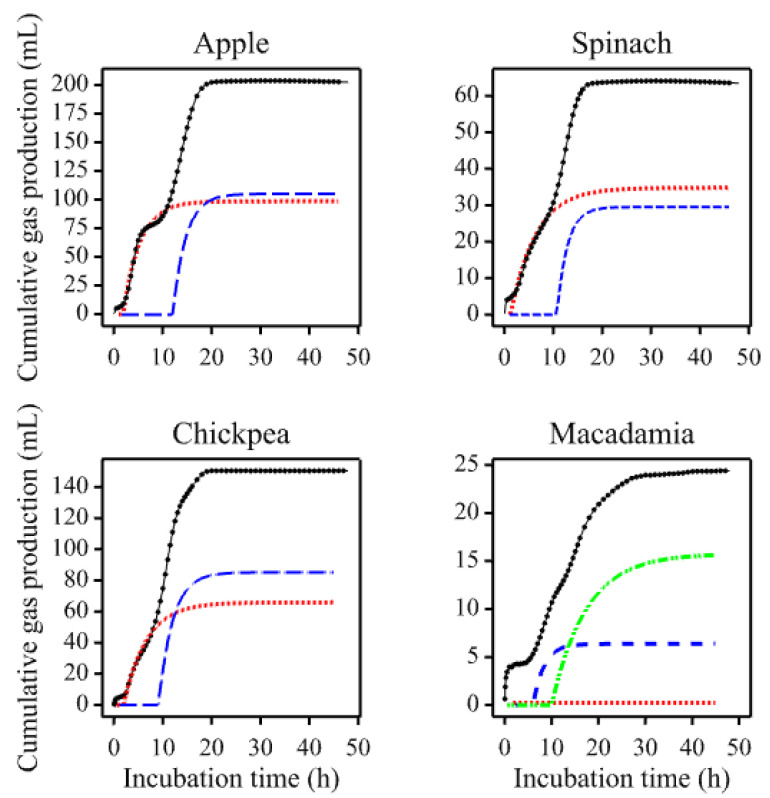
Examples (apple, spinach, chickpea, and coarsely ground macadamia) of fitting multiple Mitscherlich terms to multiphasic gas production profiles generated using porcine fecal inocula [15,16]. The figure panels show the overall fit (solid line), resolved components (broken lines), and hourly data points (dots).

Visual inspection of the 17 averaged profiles generated from the pig study [15,16] suggested they were largely multiphasic. Therefore, as a preliminary analysis, equations comprising multiple Mitscherlich terms were fitted to these profiles. Each of the seven profiles for fruit and vegetables [15] was better described using Equation (5), the double Mitscherlich ($R_{\mathrm{adj}}^{2}$ > 0.994). Again, each of the profiles for nuts and legumes (except macadamia coarse particles and the control wheat bran) [16] was better described ($R_{\mathrm{adj}}^{2}$ > 0.991) by the double Mitscherlich (Equation (5)). The macadamia profile (and that of its individual replicates) was triphasic in appearance and therefore an alternative equation with three simple Mitscherlich terms was fitted but resulted in convergence problems so an iterative curve stripping technique from pharmacokinetics was used for parameter estimation [24]. The technique, known as poly-exponential curve fitting, involved fitting a single Mitscherlich over the data range of each of the three phases, in order to produce the overall fit ($R_{\mathrm{adj}}^{2}$ = 0.967). The control wheat bran was adequately described ($R_{\mathrm{adj}}^{2}$ = 0.991) by fitting the single Mitscherlich (Equation (4)). The profiles and fitted curves obtained for apple, spinach, chickpea, and macadamia nut are illustrated in Figure 2.

### 3.2. Gas Production Curves Using Equine Fecal Inocula

**Figure 3:**
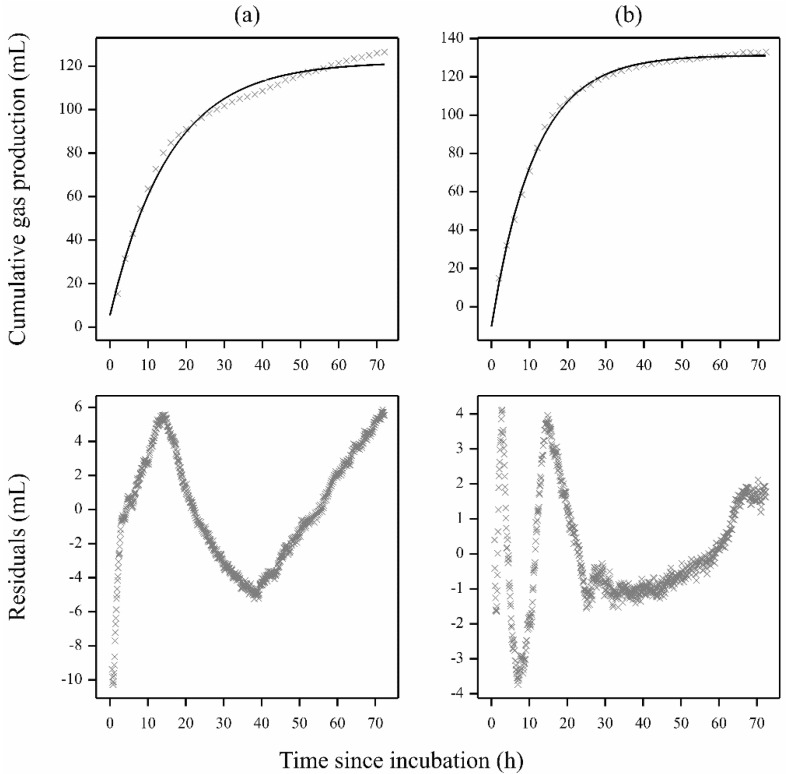
Example of monophasic gas production profiles taken from Garber et al. [19] with nondescript contaminating features: (**a**) control Treatment A (grass hay 50%, alfalfa 50%), (**b**) Treatment B (grass hay 50%, alfalfa 50%, yeast 0.011 g). The solid line shows the fitted model (simple Mitscherlich) and the crosses represent data points (only those taken every 2 h are shown with the fitted curves).

Gas production curves with equine fecal inocula may also exhibit various non-specific and non-descript features. This category of curves usually consists of a growth-curve-like base (or underlying) profile distorted by other additional features. When distortion (deviation from a monophasic pattern) is observed, fitting a standard function leaves residuals with a zig-zag pattern. This phenomenon is illustrated using data from the experiment of Garber et al. [19] and demonstrated in Figure 3 where both control and treatment data exhibit similar problems. Fitting a standard monophasic curve yields a zig-zag pattern of residuals. To absorb these features, one may use curve fitting and numerical calculations for the construction of an equivalent simple Mitscherlich profile.

**Figure 4:**
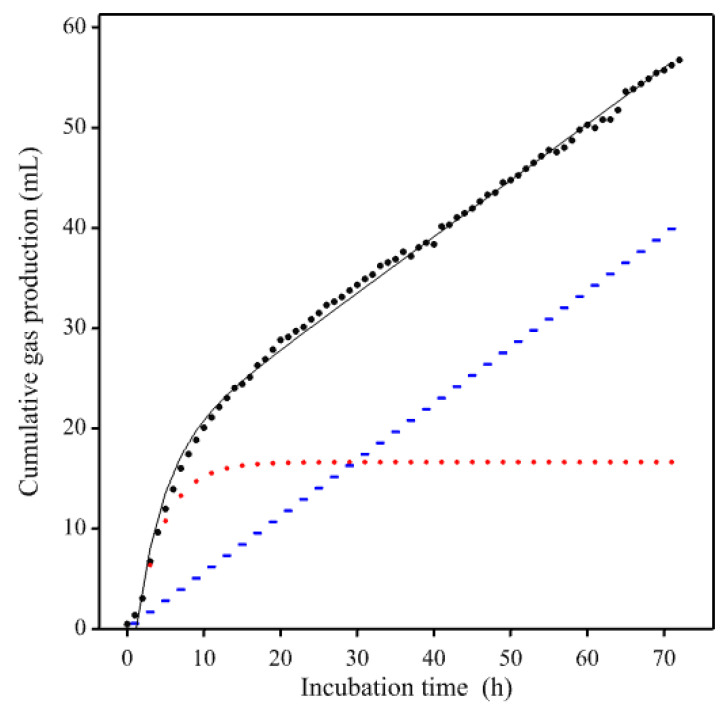
Example of a non-standard gas production curve taken from Garber et al. [19] (curve for a hay treated with 0.75 mL enzyme solution per kg dry mater and incubated with an equine fecal inoculum) showing an additional linear trend (in blue) covering the asymptotic region of the underlying growth-like curve (in red). The continuous black line shows the fitted model (a generalized Mitscherlich + linear) and the dots represent observed data points (those taken 2 hourly).

With fecal inoculum from horses and ponies, the GP profile might exhibit an additional linear trend rather than tending to an upper asymptote, as demonstrated by Powell et al. [8] who found that 8 of the 25 profiles examined were best-fitted by a biphasic Mitscherlich + linear model. Such a profile is illustrated in Figure 4 using a gas production curve from the enzyme experiment of Garber et al. [19].

**Figure 5:**
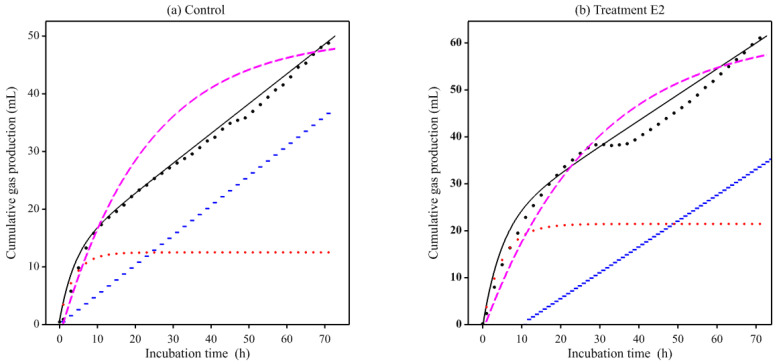
Two examples of non-standard GP profiles taken from Garber et al. [19] ((**a**) grass hay untreated; (**b**) grass hay treated with 3.75 mL enzyme solution/kg dry matter, both incubated with an equine fecal inoculum) showing an additional linear trend (in blue) covering the asymptotic region of an underlying growth-like curve (in red). The continuous black lines show the fitted (arbitrary) mathematical equation, the purple dashed lines show the constructed equivalent simple Mitscherlich equations, and the dots represent observed 2 hourly data points.

Numerical construction of a standard profile was applied using GP curves from the enzyme experiment of Garber et al. [19]. As these profiles (Figure 4) were not totally dissimilar to a diminishing returns response despite the apparently contaminating features, reconstruction was undertaken using a rectangular hyperbola plus a linear trend (i.e., $G=a_{1}/\left(1+a_{2}t^{-1}\right)-a_{3}t$, where *a*_1_ and *a*_2_ are parameters) as an appropriate mathematical equation. The data were first smoothed by spline interpolation to remove any local kinks specific to the data, the selected mathematical equation fitted and residuals computed, and then rate and relative rate calculated numerically. Rate was calculated for every pair of values of *G* and *t* as the ratio $\Delta G_{i}/\Delta t_{i}=\left(G_{i}-G_{i-1}\right)/\left(t_{i}-t_{i-1}\right)$ and the mid-point value of *G* ($\left(G_{i}-G_{i-1}\right)/2$) or *G* at $\left(t_{i}-t_{i-1}\right)/2$) was used as the divisor to calculate relative rate. To avoid undue influence of extreme values in the lower and upper quartiles, the average value from the inter-quartile range (first to the third quartile) was selected. Due to endpoint linearity, the smoothed final value was taken as the estimate of the asymptote. Similarly, lag was determined directly from the smoothed data. The results are demonstrated in Figure 5. 

**Figure 6:**
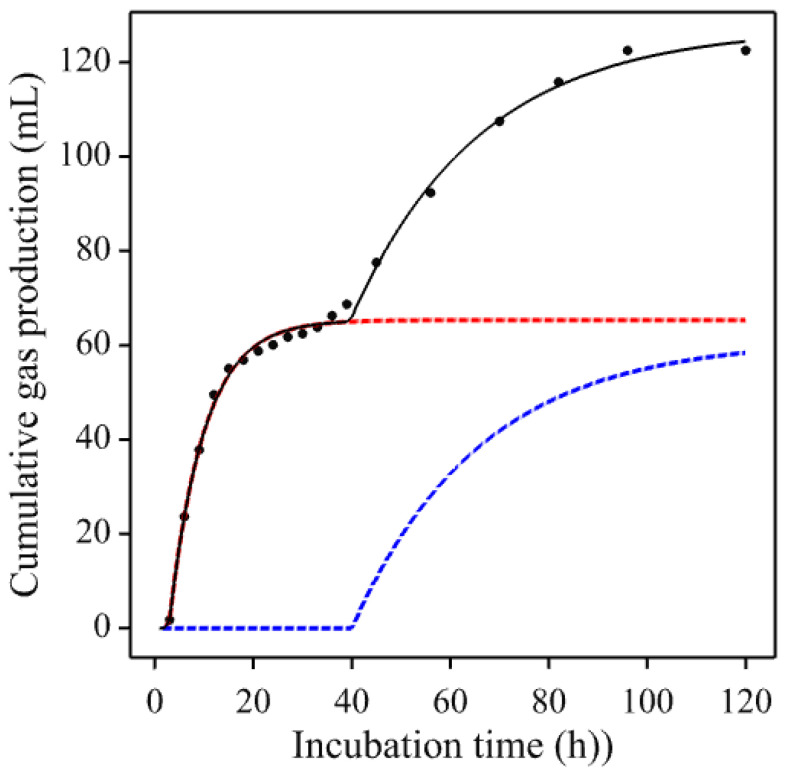
An example of fitting the simplified double Mitscherlich equation to biphasic gas production profiles generated using an equine fecal inoculum and a grass hay substrate by Murray et al. [18]. The figure shows the overall fit (solid line) and the two resolved components (red and blue broken lines). The first component starts very early in the run and approaches its asymptote, whilst the second starts much later. The dots represent observed data points.

In a first comparative evaluation, the 42 individual gas production curves (for each single replicate) of the study reported by Murray et al. [18] from 14 horses (3 replicates each) were used. Of these, 21 asymptotic biphasic profiles were identified and fitted to Equations (7) and (8). A summary of the fitting is given in Table 1 (a for Equation (7) and b for Equation (8)). From these results, it appears that the initial phase in these biphasic GP profiles was not sufficiently sigmoidal to require the more flexible double Mitscherlich (Equation (7)), and the simplified double Mitscherlich (Equation (8)) seems to be sufficient. An example of fitting Equation (8) is given in Figure 6.

A second comparative evaluation used the 17 atypical pattern (mostly biphasic) gas production profiles obtained from the averaged curves of the studies using equine fecal inocula [18,19]. From these studies, 25 gas production profiles were reported and only 8 displayed typical monophasic patterns. The six equations described above (2, 4, 5, 6, 7, and 8) were fitted to the 17 atypical profiles (each the average of 3 replicates) and the equations were compared using an array of goodness-of-fit statistics, namely adjusted *R*-square ($R_{\mathrm{adj}}^{2})$, root of residual mean square error (RMSE), and Akaike information criterion (AIC, [27]). Results of this model comparison are shown in Table 2. Residual error (RMSE) and information criterion (AIC) were lowest with the most flexible model (Equation (7)), and greatest with both monophasic Equations (2) and (4). The percentage of variance explained for by the model ($R_{\mathrm{adj}}^{2})$ was greatest with the most flexible Equation (7), followed by Equations (6) and (8). Consequently, in this comparative analysis Equation (7) showed the best performance in fitting atypical gas production profiles. Excluding this model in the comparison, Equation (8) showed the best fit for 14 profiles and Equation (6) for the other 3 curves. 

## 4. Discussion

In vitro gas production, simple as it appears, does need control of operational factors such as temperature and pressure effects on the gas volume [28]. Experimental feed or substrate requires good quality control as it undergoes processing, treatment, and preparation [29,30]. Source of inoculum (rumen liquor, feces, or fungi) will have a major effect on GP [29,31]. Feeding of donor animals (ruminants or non-ruminants) needs to be consistent with the experimental test feeds [29,30]. The atmospheric pressure and gas volumes interactions should be standardized [28] if different studies are to be compared. If one is using inoculum from animal feces, large lags can be expected before GP gets underway [2,4]. Even longer lags are seen when using fungi extracted from feces [32].

In various studies (e.g., [33]), it has been found that equine feces, as source of mixed microbial inoculum for in vitro GP, is a viable alternative to cecum-colon digesta fluid, which necessarily involves using an invasive procedure. Holter [34] found that fecal material remains largely anaerobic after voiding and the microbiota can be viable for several hours. Using the GP run-end estimates of short-chain fatty acids, substrate DM loss and GP model parameters and their functions, Lowman et al. [33] showed good correlations with in vivo DM digestibility and digestible energy. They derived regression equations for the prediction of DM digestibility (*R*^2^ = 0.75 → 0.86) and digestible energy (*R*^2^ = 0.80 → 0.88). In the equine gastrointestinal tract, inter-compartmental transit time and mean retention time (MRT) differ greatly [35]. In horses, MRT in the stomach and small intestine is on average 5 h whilst MRT in cecum-colon is on average 35 h, which is close to MRT in the rumen of ruminants [36]. Pre-hindgut digestion does not degrade the structural carbohydrates (fiber) of the feed, which undergoes microbial fermentation in the cecum-colon ecosystem [37,38]. In the case of fecal-based inoculum, an extent of digestion calculation may perhaps over-adjust for passage losses because lag-time estimates from non-ruminal inocula tend to be longer, possibly due to the extra time required for the microbial population to achieve an optimum level. Thus, it might not be strictly appropriate to impose the passage losses concept to feedstuff degradation in hindgut fermenter or non-ruminant herbivores. Extent in equines for example is likely to be associated mainly with the cecum-colon compartment.

The analysis of standard GP curves is now well advanced using the in vitro GP model of France et al. [14] (generalized or simple Mitscherlich) together with other growth or enzyme kinetic functions in their classical or modified forms (e.g., [39]). Furthermore, France et al. [40] and Powell et al. [8] linked in vitro GP results to events in the rumen or cecum-colon proper, given some estimate of digesta rate of passage from the relevant compartment. If in vitro GP is from substrate derived from animal feed matter, then one should be interested in the in vivo digestibility and account for losses due to particle passage rate from (say) the rumen or cecum-colon.

Cumulative gas production curves may exhibit atypical profiles deviating from the most commonly expected growth-like profiles, especially when fecal matter is used as inoculum for the incubations. Curve smoothing can be used as an initial step to remove any noise or perturbation that could hidden the underlying trend. In our work, smoothing has been used to visualize the nature of these atypical curves. In the example with an exponential + linear trend (Figure 4), numerical calculations using splines regression with large degrees of freedom were used for the smoothing. Without smoothing, distribution of fermentation rate (d*G*/d*t*) values showed some extreme values, whilst after smoothing rate values were much more as expected. The reason for the linear trend may arise from the inoculum either due directly to the treatment (or any known modification) or some unknown interaction therein. Therefore, further analysis of the inoculum may be necessary. Residuals for the reconstructed trend can be allocated from the initial fit of an appropriate but arbitrary mathematical equation. However, to avoid importing contaminating features, this allocation should be made at random prior to any further analysis.

Analysis of atypical GP curves is the topic of this work, as little information is available in the published literature. Biological responses may consist of two or more components which may be overlapping, convoluted, or sufficiently separated as seen in biphasic GP profiles. To describe multiphasic profiles, one can construct models from simpler standard functions as described by Powell et al. [8] and herein for the simple Mitscherlich equation. The biphasic models (Equations (5)–(8)) were found to describe the atypical gas production profiles accurately, providing a better performance (in terms of goodness-of-fit) than monophasic Equations (2) and (4). This is in agreement with the results reported by Powell et al. [8], who found that biphasic equations were more suitable to describe atypical GP profiles from cultures inoculated with equine feces. Multiphasic models have been used [5,6,7] to describe GP curves from in vitro batch cultures inoculated with ruminal fluid. It is well worthy to mention that in those studies, the multiphasic models were applied to typical GP profiles intending to represent the differential fermentation rates of the diverse feed fractions (soluble and readily fermentable or insoluble fiber slowly fermented), or the fermentation of microbial matter once the potentially fermentable substrate is exhausted [6,7]. However, there are very few studies reporting the fit of atypical curves, which may occur more frequently when fecal inocula are used. Powell et al. [8] reported a first comparison between mono- and bi-phasic models for this sort of atypical GP profile. To facilitate nonlinear parameter estimation, models should be parsimonious (i.e., as few parameters as possible). However, it was clearly demonstrated that a simple or generalized monophasic Mitscherlich can be insufficient to describe atypical curves. The generalized equation derived by France et al. [14] is more flexible than the simple monomolecular, representing a sigmoidal pattern with a variable point of inflexion. However, both functions require that the asymptote is well defined, otherwise they may result in unsatisfactory fits. In fact, both equations are outcompeted by biphasic models to fit atypical GP curves as shown by Powell et al. [8], and confirmed in the comparisons reported herein. In our study, the comparisons were focused exclusively on atypical curves. It has been shown that the more generalized biphasic Equation (7) provided the best fit for average curves, but the double exponential (Equation (8)) can be a suitable candidate. The fact that the double-exponential model performed better in one comparison, but the most flexible equation showed better goodness-of-fit in the other, indicates that the selection of a model to describe these atypical curves needs to be made on a case-by-case basis in order to make data compatible with the attributes of the chosen function. Nevertheless, the use of multiphasic models may still be not enough in some cases, where curve smoothing might be required to make data more compatible with the chosen function attributes. By using appropriate mathematical equations, curve peeling, and numerical calculations, the construction of an equivalent growth-like profile can be undertaken. After fitting a suitable mathematical equation, one can isolate any contaminating trends in the residuals. In order to avoid transferring these trends into the new curve the residuals can be allocated at random to the reconstructed underlying trend (e.g., using RANDOM.ORG to generate random numbers [23]). For this process, a response function with constant relative rate such as the Mitscherlich equation is ideal.

## 5. Conclusions

Analysis of atypical gas production profiles obtained using a fecal inoculum is illustrated. Atypical gas production profiles are characterized by the presence of multiple phases or non-descript extraneous features which make it difficult to apply directly recommended modeling approaches such as standard response functions or classical growth functions. To overcome such difficulties, extensions of the Mitscherlich equation and a numerical modeling option also based on the Mitscherlich are proposed and illustrated. Due to their hybrid nature, the models promulgated described the atypical curves well. These models contain kinetic parameters that can be used to calculate extent of substrate degradation and, given that extent of degradation is linked to nutrient supply, they provide useful information regarding the evaluation of feedstuffs using non-invasive in vitro methods.

## Figures and Tables

**Table 1:** Analysis of biphasic profiles [18] using either (**a**) a transformed double Mitscherlich or (**b**) the simplified transformed double Mitscherlich as the gas production model (summarized results for the 21 asymptotic biphasic profiles identified).

	Residual SD ^§^	$R_{adj}^{2}$ (%) ^§^	Runs Test	Concordance *	SD Ratio ^†^
(a) Equation (7)					
Mean	5.94	96.1	7.7	0.989	2.294
Median	4.93	96.7	7	0.991	1.754
Minimum	3.15	85.7	6	0.955	1.152
Range	16.95	13.1	5	0.041	6.459
*q*_1_^§^	3.59	96.1	7	0.987	1.443
*q*_3_^§^	6.03	98.1	8.25	0.995	2.888
SEM ^§^	0.864	0.688	0.326	0.002	0.318
(b) Equation (8)					
Mean	2.75	99.0	8.7	0.997	0.542
Median	2.33	99.4	8	0.998	0.570
Minimum	1.80	97.2	7	0.990	0.131
Range	4.11	2.70	5	0.0096	0.737
*q*_1_^§^	1.99	98.3	7	0.995	0.347
*q*_3_^§^	3.06	99.6	10.25	0.998	0.693
SEM ^§^	0.237	0.164	0.380	0.0006	0.045

^§^ SD = standard deviation; $R_{\mathrm{adj}}^{2}$ = adjusted *R*-square; *q*_1_= lower and *q*_3_ = upper quantile; SEM = standard error of the mean. * Concordance index of Lin [25] measures closeness of fit to the data profile. ^†^ 95% confidence interval (CI) multiplicative factors are 0.64 and 1.57. If 1.0 is outside the CI, then the numerator SD is significantly different from the denominator SD, (this ratio is calculated as full model SD/simplified model SD) [26].

**Table 2:** Goodness-of-fit of the six equations (two monophasic [Equations (2) and (4)] and 4 biphasic [Equations (5)–(8)]) to 17 atypical average gas production profiles obtained in incubations using equine fecal inocula.

		Equation (2)	Equation (4)	Equation (5)	Equation (6)	Equation (7)	Equation (8)
Parameters		4	3	6	5	6	5
RMSE ^§^							
	Mean	5.97	6.35	4.80	4.59	1.92	4.13
	Median	6.34	6.96	4.64	4.94	2.06	4.46
	Minimum	1.73	2.18	2.33	0.76	0.43	0.95
	Maximum	10.74	9.10	8.37	6.70	3.64	7.09
$R_{\mathrm{adj}}^{2}$ ^§^							
	Mean	94.6	94.0	96.3	97.0	99.3	97.5
	Median	95.4	94.6	97.1	97.0	99.6	97.4
	Minimum	83.8	86.7	90.2	94.0	97.3	93.0
	Maximum	99.0	98.3	98.5	99.8	99.9	99.7
AIC ^§^							
	Mean	70.6	72.8	66.8	55.0	20.0	51.7
	Median	72.4	75.3	63.9	63.5	30.8	59.6
	Minimum	36.5	50.0	49.1	−13.8	−47.8	−0.15
	Maximum	92.4	85.4	98.0	75.1	52.3	77.2

**^§^** RMSE = root mean square error; $R_{\mathrm{adj}}^{2}$= adjusted *R*-square; AIC = Akaike information criterion.

## Data Availability

The data presented in this study are available in the articles cited in the text.
